# A statistical analysis plan for the efficiency and safety of Chinese herbal medicine used concurrently with topical therapy for psoriasis vulgaris

**DOI:** 10.1186/s13063-016-1610-z

**Published:** 2016-10-03

**Authors:** Liming Lu, Meiling Xuan, Yuhong Yan, Geng Li, Li Zhou, Zehuai Wen, Chuanjian Lu

**Affiliations:** 1 Key Unit of Methodology in Clinical Research, Guangdong Provincial Hospital of Chinese Medicine, Guangzhou, 510120 China; 2 Department of Dermatology, Guangdong Provincial Hospital of Chinese Medicine, Guangzhou, 510120 China; 3 National Center for Design Measurement and Evaluation in Clinical Research, Guangzhou University of Chinese Medicine, Guangzhou, 510405 China

**Keywords:** Psoriasis vulgaris, Randomized controlled trial, Calcipotriol Betamethasone, Calcipotriol, YXBCM01 granule, Chinese medicine, Statistical analysis plan

## Abstract

**Background:**

Psoriasis vulgaris (PV) has been causing increasing concern due to its highly prevalent, harmful and therapy-resistant characteristics. The YXBCM01 (Chinese herbal medicine) for PV trial evaluates the effects of YXBCM01 on relapse rate in patients suffering from PV. As an update to the published design and method for the trial, this paper presents the statistical plan for the main publication to avoid the risk of outcome reporting bias, selective reporting, and data-driven results.

**Methods/design:**

This trial is a multicenter, randomized, double-blind, placebo-controlled, parallel-group clinical trial. A total of 600 PV patients (300 in each group) will be randomized to one of two arms: participants in the experimental group will receive the YXBCM01 granule 5.5 g twice daily for 12 weeks. Placebo granules are given to patients in the control group at a dose of 5.5 g twice daily for 12 weeks. The sequential topical therapy is administrated simultaneously to all eligible patients by using calcipotriol betamethasone ointment once daily (a treatment area of up to 30 % body surface area (BSA), fingertip unit is recommended) in the first 4 weeks (maximum of 100 g weekly), followed by calcipotriol betamethasone ointment once daily for the remaining 8 weeks (maximum of 100 g weekly). The primary outcome measure is relapse rate in the treatment period and follow-up period. The secondary outcome measures include time to relapse, time to onset, rebound rate, cumulative consumption of topical medicine, visual analog scale (VAS), BSA, the Dermatology Life Quality Index (DLQI) and the Medical Outcomes Study (MOS) 36-item short form health survey (SF-36).

**Conclusions:**

Application of this statistical analysis plan to the YXBCM01 for PV trial will facilitate unbiased evaluation of these important clinical data. This study will provide evidence regarding the value of YXBCM01 as an intervention for PV patients.

**Trial registration:**

Chinese Clinical Trial Registry: ChiCTR-TRC-13003233, registered on 26 May 2013.

**Electronic supplementary material:**

The online version of this article (doi:10.1186/s13063-016-1610-z) contains supplementary material, which is available to authorized users.

## Background

Psoriasis vulgaris (PV) is an immunologically abnormal, chronic, proliferative dermatopathy with polygenic inheritance [1]. It is causing increased concern due to its highly prevalent, harmful and therapy-resistant characteristics in China and other countries [2].

Chinese herbal medicine (CHM) for the treatment of psoriasis has accumulated a wealth of clinical experience in recent years [3–6]. However, there is no evidence of benefit from CHM for PV relapse. The use of a Chinese herbal compound, YXBCM01, composed of *Radix Paeoniae Rubra* (*Chishao*), *Sarcandra Glabra* (*Jiujiecha*), *Rhizoma Smilacis Glabrae* (*Tufuling*), etc., supposes that there is an effect on reducing PV relapse based on Chinese medicine theory and clinical observations [2, 7]. The present study is designed to examine the efficacy and safety of YXBCM01 for PV in a clinical trial. The protocol of this research has been published in *Trials* [2].

To prevent outcome reporting bias and data-driven analysis results, the International Conference on Harmonization (ICH) guidelines state that the primary analysis should be prespecified [8]. Here, we present the statistical analysis plan containing the trial’s main results while the data collection in this trial is still ongoing. Full details regarding the rationale and design of the study are given in the study protocol.

## Methods/design

### Study design

The present study is designed to examine the efficacy and safety of YXBCM01 for PV in a multicenter, randomized, double-blind, placebo-controlled, parallel-group clinical trial. Eleven general or dermatological hospitals in different provinces in China will participate in the study: Guangdong Provincial Hospital of Chinese Medicine, Guangzhou; China-Japan Friendship Hospital, Beijing; Longhua Hospital, Shanghai University of TCM, Shanghai; Xinjiang Uygur Autonomous Region Hospital of Traditional Chinese Medical, Urumqi; the First Affiliated Hospital of Guangzhou University of Chinese Medicine, Guangzhou; the Affiliated Hospital of Chengdu University of Chinese Medicine, Chengdu; Guang’anmen Hospital, China Academy of Chinese Medical Sciences, Beijing; Beijing Hospital of Traditional Chinese Medicine, Beijing; Heilongjiang Academy of Traditional Chinese Medicine, Harbin; Wuhan Integrated Traditional Chinese Medicine and Western Medicine Hospital, Wuhan; and the Third Hospital of Hangzhou, Hangzhou. This trial was approved by six sites’ ethics committees and required archival filing management with five other sites’ ethics committees (Additional file 1). Results of this study will provide evidence regarding the value of YXBCM01 as an intervention for PV patients.

### Trial population and eligibility

A total of 600 PV patients (300 in each) will be enrolled in our trial. The inclusion and exclusion criteria are presented in Table 1. Eligible patients will be randomized to the experimental and control groups. Participants in the experimental group will receive the YXBCM01 granule 5.5 g twice daily for 12 weeks. Placebo granules are given to patients in the control group at a dose of 5.5 g twice daily for 12 weeks, the main ingredients of which, maltodextrin, lactose and natural edible pigment, will be identical to the YXBCM01 granule in appearance, weight and taste. The sequential topical therapy is administrated simultaneously in all eligible patients by using calcipotriol betamethasone ointment once daily (treatment area up to 30 % body surface area (BSA), fingertip unit is recommended) in the first 4 weeks (maximum of 100 g weekly), followed by calcipotriol betamethasone ointment once daily for the remaining 8 weeks (maximum of 100 g weekly) [9], which is recommended by the S3-Guidelines for the Treatment of PV [10].

**Table 1:** Inclusion and exclusion criteria for Chinese herbal medicine regarding complications of topical therapy for psoriasis vulgaris (PV)

Inclusion	Patients with a clinical diagnosis of a psoriatic plaque (diagnosis of PV is compliant with guidelines of care for the management of psoriasis and psoriatic arthritis, 2008 [11, 10]) can be enrolled into this study if they:
	1. Are ≥18 to ≤65 years of age
	2. Have a PASI score >10 or body surface area (BSA) >10 %
	3. Have a PASI score <30
	4. Have a BSA <30 %
	5. Have provided written informed consent from self or a legal surrogate
Exclusion	Patients are excluded from the study if any of the following criteria apply:
	1. Presence of guttate psoriasis, inverse psoriasis or psoriasis exclusively involving the face
	2. They are pregnant, lactating, or plan to become pregnant within a year
	3. They have a Self-rating Anxiety Scale (SAS) score >50 or a Self-rating Depression Scale (SDS) score >53, or other psychiatric disorders
	4. Have uncontrolled cardiovascular, respiratory, digestive, urinary, or hematological disease
	5. They have known cancer, infection, electrolyte imbalance, acid-base disturbance, or calcium-related metabolic disorder
	6. There is an abnormal serum calcium level (Ca^2+^ >2.9 mmol/L or <2 mmol/L)
	7. They are allergic to any medicine or ingredients used in this study
	8. They are currently enrolled in other clinical trials or participated in one within the previous 1 month
	9. They have used topical treatments (i.e., corticosteroids, retinoic acid) within the previous 2 weeks; systemic therapy or phototherapy (ultraviolet B radiation, UVB) and psoralen (combined with ultraviolet A radiation, PUVA) within the previous 4 weeks; or biological therapy within the previous 12 weeks
	10. They have acute progression of psoriasis and an erythrodermatitic tendency
	11. They are patients who need systemic treatment prescribed by a physician

*PASI* Psoriasis Area Severity Index

The protocol was approved by all sites’ ethics committees or required archival filing management with the sites’ ethics committees. Research assistants will obtain a signed consent form from all patients who are willing to participate in the trial.

### Sample size

Based on White’s study [9], the relapse rate of sequential topical therapy at 12 weeks is 37.3 %, and placebo is 46.6 %. From this, supposing the relapse rate of the YXBCM01 granule combined with sequential topical therapy for PV in the 12th week is 20 %, sequential topical therapy alone is 37 %. According to this supposition, and calculated by PASS 11.0 software, a total sample size of 478 in the two groups can achieve 90 % power and rule out a two-sided type I error of 5 % to detect a superiority margin difference of 5 % in this two-arm trial, with an allocation ratio of 1:1. Considering 15 % loss to follow-up and other factors in numbers of centers, the total sample size should be adjusted to 600 in the two groups.

### Randomization and blinding

A total of 600 participants will be enrolled from eligible patients at 11 sites. A computer- generated random list for the center-stratified method and permuted block size created by SAS 9.2 software (SAS Institute Inc., Cary, NC, USA) will be used for randomization. The randomization list and blinding codes will be performed by the Key Unit of Methodology in Clinical Research (KUMCR) of Guangdong Provincial Hospital of Chinese Medicine. Blinding was ensured using a matched placebo granule identical in color, size, shape and taste.

### Data sets

Efficacy analyses will be performed for both the intent-to-treat (ITT) population and the per-protocol (PP) population. The ITT population will consist of all randomized subjects. In a PP analysis, only patients who complete all or most of the clinical trial according to the protocol are counted towards the final results. Besides, the PP population in our research will meet these criteria: the experimental treatment requirements; the main variables can be determined; no missing baseline variables; no major violation of test scheme; and have taken the total treatment dosage with a compliance of 80–120 %. Safety analyses will be performed on the safety population, which will be comprised of all randomized subjects who have been administered at least one treatment. When the PP and ITT analysis conclusions are not the same, it is necessary to analyze the possible reasons and discussion them. All the variables, measures and methods of statistical analysis are presented Table 2.

**Table 2:** Variables, measures and methods of analysis

Variable/outcome	Hypothesis	Outcome measure	Methods of analysis
1. Primary	Intervention improved outcome from baseline to follow-up period		
Relapse rate	Improvement occurred	Questionnaire (binary)	Chi-squared test
2. Secondary			
Time to relapse	Improvement occurred	Questionnaire (time to event)	Log-rank test
Rebound rate	Improvement occurred	Questionnaire (binary)	Chi-squared test
PASI	Improvement occurred	Questionnaire (continuous)	*t* test/ANOVA
DLQL	Improvement occurred	Questionnaire (continuous)	*t* test/ANOVA
VAS	Improvement occurred	Questionnaire (continuous)	*t* test/ANOVA
SF-36	Improvement occurred	Questionnaire (continuous)	*T* test/ANOVA
Time to onset	Improvement occurred	Questionnaire (time to event)	Log-rank test
Cumulative consumption of topical medicine	Improvement occurred	Recorded amount (continuous)	*t* test/ANOVA
SAS	Improvement occurred	Questionnaire (continuous)	*t* test/ANOVA
SDS	Improvement occurred	Questionnaire (continuous)	*t* test/ANOVA
BSA	Improvement occurred	Questionnaire (continuous)	*t* test/ANOVA
Direct medical cost	Improvement occurred	Recorded amount (continuous)	*t* test/ANOVA
3. Subgroup analyses			
Severity of disease	Improvement occurred		Chi-squared/*t* test/ANOVA
Chinese medicine patterns	Improvement occurred		Chi-squared/*t* test/ANOVA
4. Sensitivity analyses	Improvement occurred	All outcomes	
Per-protocol analysis			Chi-squared/*t* test
Adjusting for baseline covariates			Multivariable regression/Cox proportional hazards model
clustering among individuals within a hospital			Generalized estimating equations/Cox proportional hazards model
5. Safety analyses			
Frequency of adverse events (AEs)	Improvement occurred	Recorded amount (binary)	Chi-squared test
Cases divided into different degree of AE	Improvement occurred	Recorded amount (binary)	Rank-sum test
6. Other analyses			
Compliance rate		Recorded amount (binary)	Chi-squared test
Analysis of drug combinations		Recorded amount (binary)	Descriptive statistics
Analysis of shed-off cases		Recorded amount (binary)	Descriptive statistics

*ANOVA* repeated measure analysis of variance, *BSA* body surface area, *DLQL* Dermatology Life Quality Questionnaire, SAS self-rating anxiety scale, *SDS* self-rating depression scale, *SF-36* the MOS 36-item short form health survey, *PASI* Psoriasis Area Severity Index, *VAS* visual analog scale

### Demographic and baseline characteristics

Demographic data include, but are not limited to, sex, age, educational level, occupation, marital status, ethnicity, allergic history and family history. Baseline characteristics include, but are not limited to, respiratory frequency, heart rate, pulse, blood examination, and questionnaire scores. Comparisons between the experimental group and the control group will be conducted to assess the degree to which comparability of randomization was achieved.

### Primary outcome analyses

The Psoriasis Area Severity Index (PASI) will be assessed every week during the first 4 weeks and every 2 weeks throughout the rest of the treatment and follow-up period (the higher the PASI, the worse the treatment effect). The primary outcome measure in the trial is the relapse incidence rate in the treatment period and the follow-up period. The definition of relapse is a loss of 50 % of PASI improvement from baseline in patients who have achieved treatment success (at least 50 % improvement in PASI score from baseline) [11].

The primary outcome relapse rate between the two groups will be compared by the chi-squared test. Frequency counts and percentage of subjects in each arm will be provided for categorical data.

### Secondary outcome analyses

Secondary outcome measures include time to relapse, time to onset, rebound rate, cumulative consumption of topical medicine, visual analog scale (VAS), body surface area (BSA), the Dermatology Life Quality Index (DLQI) and the SF-36 (the Medical Outcomes Study (MOS) 36-item short form health survey). Time to relapse is the duration between improvement (PASI score decreases more than 50 % from baseline, PASI-50) and recurrence appearance for the first time (loss of at least 50 % of the PASI improvement). Time to onset is a PASI score decrease of more than 50 % for the first time, which will be considered as ineffective when the PASI score cannot achieve 50 % improvement throughout the treatment period. Rebound (a binary variable) refers to a PASI score increase of more than 125 % above baseline, or the occurrence of new generalized pustular and erythrodermic lesions after its initial improvement (PASI-50) during the study period. VAS and BSA will be assessed every week during the first 4 weeks and every 2 weeks in the rest of the treatment period. DLQI and SF-36 will be assessed every 4 weeks throughout this trial.

The rebound rate between the two groups will be compared by the chi-squared test. Time to relapse and time to onset will be analyzed using a log-rank test. Other secondary outcomes are continuous variables, thus they will be summarized with *n*, mean, standard deviation and range if they are approximately normal and with *n*, median, interquartile range and range if non-normal (for example, skewed). At one time point, comparisons between the experimental group and the control group will be conducted using a *t* test. In order to better distinguish the treatment effect and time effect, the changes from baseline to the endpoint of treatment in the above outcomes will be tested with a repeated measure analysis of variance (ANOVA).

### Subgroup analyses

We plan to conduct two subgroup analyses, both with strong pathological associations and possible interaction effects. The first will compare the primary and secondary outcomes (relapse rate, time to relapse, PASI, DLQL, VAS, SF-36, time to onset, cumulative consumption of topical medicine, SAS, SDS, BSA and direct medical cost) based upon the degree of disease severity. Patients will be classified into four subgroups by PASI scores of 10–15, 16–20, 21–25, 26–30 at baseline. The second will compare the primary and secondary outcomes among different Chinese medicine patterns. In Chinese medicine, PV is thought to be due to blood heat, dryness, blood deficiency, blood stasis and other causes. We will divide the patients into the subgroups of blood heat, dryness, blood deficiency, blood stasis and others according to the standard of Chinese medicine patterns. We will use ANOVA for continuous outcomes for three or more subgroups, and the chi-squared test for binary outcomes.

### Sensitivity analyses

Per-protocol analysis is presented as the primary and secondary efficacy analyses of ITT analysis. At one time point, a *t* test is used for continuous outcomes and the chi-squared test for the binary outcomes. ANOVA is used for testing the changes at different time points between the experimental and control groups.

Adjusting for baseline covariates, for timed endpoints, such as time to relapse and time to onset, we will use the Kaplan-Meier survival analysis followed by the multivariable Cox proportional hazards model for adjusting for baseline variables. For continuous outcomes, such as PASI, DLQL, VAS, SF-36, cumulative consumption of topical medicine, SAS, SDS, BSA and direct medical cost, a linear regression model will be used to control the baseline covariates. For the binary outcome relapse rate, a logistic regression model will be performed for controlling baseline covariates.

To assess the impact of potential clustering for patients cared for by the same hospital, we will use a Cox proportional hazards model for the outcome of time to relapse or time to onset, and generalized estimating equations (GEE) for other outcomes with center as a random effect.

### Safety analyses

Participants are to be questioned about adverse events (AEs) during the study at each visit point, and all AEs reports will be recorded and assessed by the investigators for a causal relationship with the treatment. Blood tests, urinalysis, a hemagglutination test and electrocardiographic examination will be checked before and after the treatment. Furthermore, calcium, kidney and liver function tests will be reexamined at week 4 and week 12, respectively. All abnormal changes from baseline laboratory tests will be evaluated by the investigators.

Safety will be evaluated by tabulations of AEs and will be presented with descriptive statistics at baseline and follow-up visits for each treatment group. The statistics will be organized by treatment phase and post-treatment phase as appropriate. Adverse event incidence rates will be summarized by organ system class, preferred term, and severity of the AE. Each subject will be counted only once within an organ system class or a preferred term by using the AEs with the highest severity within each category. Adverse events will also be summarized by organ system class, preferred term, and relationship to procedure. Each subject will be counted only once within an organ system class or a preferred term by using the AEs with the closest relationship to treatment within each category.

All information pertaining to AEs noted during the study will be listed by subject, detailing verbatim by the investigator: preferred term, organ system class, date of onset, date of resolution, severity, and relationship to procedure.

A tabulation of serious adverse events (SAEs) will be provided by subject within treatment groups. The chi-squared test or Fisher’s exact test will be used to compare the frequency difference of AEs between the experimental and control groups. As cases are divided into different degree of AEs, a rank-sum test will be performed for analyzing the independent ordered multiple category data between the two treatment groups.

### Other analyses

Compliance rates are divided into three categories, such as below 80 %, 80–120 % and above 120 %. We will use the chi-squared test to detect the difference in compliance rates between the two treatment groups. Comparison of concomitant drug usage (number of subjects using traditional Chinese medicine or modern medicine) and shed-off cases will be executed by descriptive statistics between the two treatment groups.

### Handling of missing data

In the event of nonresponse after each follow-up, the subjects will be informed by our researchers and asked to provide the missing data. Even with the reminders, some loss to follow-up is expected over 12 weeks. The proportion of participants missing each outcome will be summarized in each arm and at each time point.

If there is less than 5 % of data missing for a specified primary or secondary outcome, we will perform a complete case analysis without imputing the missing values. If there is more than 5 % of data missing we will perform Little’s test. We will continue analyzing the complete cases without imputing missing values if the complete case data set is indicated by Little’s test to be a random sample. We will report the point estimates and their 95 % confidence interval (CI) applying a worst- and best-case scenario imputation for the missing values if the complete case data set is not indicated by Little’s test to be a random sample. Multiple imputations will not be performed if the worst- and best-case analyses allow for the same conclusion. Otherwise, multiple imputations will create 10 imputed data sets under the assumption of data missing at random. The results of the trial will be the pooled intervention effect and 95 % CI of the analyses of each data set after multiple imputation.

#### Current status

The trial commenced in April 2014 at 11 hospitals in China. Enrollment has been ongoing. The final participant will be followed up in December 2017.

## Conclusions

The present study is designed to examine the efficacy and safety of YXBCM01 for PV in a multicenter, randomized, double-blind, placebo-controlled, parallel-group clinical trial. Application of this statistical analysis plan to the trial will facilitate unbiased evaluation of these important clinical data and support confidence in the subsequent generalization of its findings.

## Additional file

Additional file 1: List of approvals from six hospitals’ ethics committees and archival filing management required in five hospitals’ ethics committees. (DOC 26 kb)

## Abbreviations

AEs: Adverse events
ANOVA: Repeated measure analysis of variance
BSA: Body surface area
CHM: Chinese herbal medicine
CI: Confidence interval
DLQI: The dermatology life quality index
GEE: Generalized estimating equations
GMP: Good Manufacturing Practice
ICH: The International Conference on Harmonization
ITT: Intent-to-treat
PASI: The Psoriasis Area Severity Index
PASI-50: PASI score decreases more than 50 % from baseline
PP: Per-protocol
PUVA: Psoralen combined with ultraviolet A
PV: Psoriasis vulgaris
SF-36: The MOS item short form health survey
SAEs: Serious adverse events
SAS: Self-rating anxiety scale
SDS: Self-rating depression scale
VAS: Visual analog scale
